# Improved Pregnancy Outcomes in Beef Heifers Through Delayed Insemination of Sexed Semen

**DOI:** 10.1111/rda.70111

**Published:** 2025-08-18

**Authors:** R. Kasimanickam, K. Ratzburg, K. Madsen, R. Keckler

**Affiliations:** ^1^ Department of Veterinary Clinical Sciences College of Veterinary Medicine, Washington State University Pullman USA; ^2^ Marias Veterinary Clinic Shelby Montana USA; ^3^ Mountain View Veterinary Service Twin Bridges Montana USA; ^4^ Animal Health Clinic Blackfoot Idaho USA

**Keywords:** beef heifers, economics, estrous response, gender ratio, insemination, pregnancy, sexed semen

## Abstract

This study evaluated the effect of the timing of Y‐sorted sexed semen (SS) insemination after the onset of estrus on reproductive outcomes in beef heifers and examined the influence of AI sires and their sperm DNA fragmentation (%SDF) over time. Angus heifers (*n* = 718) from two locations were synchronised using a CIDR + Select‐Synch protocol and blocked by age, body condition score, and reproductive tract score. Heifers expressing estrus were randomly assigned to AI at 12, 20, or 28 h post‐estrus onset using SexedULTRA 4 M semen from one of three bulls. Post‐thaw %SDF at 0, 12, and 24 h was assessed by acridine orange staining. Pregnancy per AI (P/AI) differed by timing: 12 h (45.4%), 20 h (48.1%), and 28 h (56.5%) (*p* < 0.05), with the 28 h group achieving significantly higher P/AI than the 12 h group. Stillbirth incidence and gender ratio (bull: heifer) did not differ significantly among groups. No overall difference in P/AI among sires was observed; however, a significant sire × time of AI interaction existed (*p* = 0.05), with Bull 2 showing the greatest improvement in P/AI at 28 h (up to 15.2 percentage points higher than earlier times). For Bulls 1 and 2, %SDF increased significantly from 0 to 24 h post‐thaw, while Bull 3 showed no change. These findings indicate that delaying insemination to 28 h post‐estrus enhances P/AI when using Y‐sorted SS, potentially due to improved synchrony with ovulation and reduced exposure to sperm with increasing DNA damage.

## 1 Introduction

Sexed semen (SS) has been designed for the thoughtful production of calves of the desired sex. The acceptance and usage of AI in the US is much less (11.6%) in the beef production system (USDA 2020). The SS is more expensive, and it results in reduced pregnancy rates compared to conventional semen (Thomas et al. 2017). Therefore, the use of SS in the beef production system is also low. The beef industry will profit from the application of SS when the monetary return from producing offspring of a desired sex is greater than the cost of implementing it (Hohenboken 1999). Nevertheless, SS with high fertility could transform beef production. However, an obvious limitation is that insemination with SS results in conception rates ranging from 75% to 90% of conventional semen (CS), which is a 5 to 10 percentage points decrease in pregnancy per AI (P/AI) (Maicas et al. 2019; Seidel Jr. 2014). Initially, SS was recommended for inseminating nulliparous heifers 12 h after a detected estrus (Maicas et al. 2019; Seidel Jr. et al. 1999; Seidel Jr. 2014). However, delayed insemination with SS at 16 + h (Nebel 2018), and between 23 and 41 h (Bombardelli et al. 2016) relative to the onset of standing estrus increased P/AI in Jersey cattle. Though the timing of AI using SS has been evaluated recently within the context of a fixed‐time synchronisation programme, using hormones to synchronise ovulation, in which the timing of AI relative to the induction of ovulation is precisely controlled, still there has been a huge variation in P/AI considering the timing of the onset of estrus and the timing of insemination with SS (Hall et al. 2017; Kasimanickam et al. 2021). In beef cattle, ovulation occurs approximately 32 h after the onset of estrus, and insemination with CS between 4 and 20 h after the onset of estrus resulted in acceptable P/AI (Dorsey et al. 2011; Stevenson et al. 1996; White et al. 2002). The optimal interval from ovulation to insemination is vital to allow adequate time for sperm transport and maturation in the female reproductive tract relative to ovulation. Given that oocyte lifespan is constant, 6 to 12 h after ovulation (Brackett et al. 1980), the time required for sperm transport in the female reproductive tract, maturation and capacitation (Wilmut and Hunter 1984) relative to ovulation could be impacted by changing the timing of insemination.

Sperm sorting technology successfully separates X‐ and Y‐chromosome‐bearing sperm based on differences in DNA content to produce sperm units for commercial use in cattle (Garner et al. 1983; Saacke et al. 2000). Sex‐sorted sperm produced using this method have reduced viability and overall quality after cryopreservation and thawing due to molecular aberrations including damage to sperm DNA (Boe‐Hansen et al. 2005; Gosálvez et al. 2011) and have led to a poor fertilisation rate (Steele et al. 2020). Recent research efforts have resulted in the development of next generation technology with optimised equipment and procedures that decrease the degree of sperm damage occurring during the sex‐sorting process (DeGraaf et al. 2014; Evans 2010; Sharpe and Evans 2009). Sexed semen produced using this new technology is presently available and marketed commercially under the trade name SexedULTRA 4 M. A large field trial (8 bulls; over 6000 dairy heifers) using SS insemination based on detected estrus indicated 4.5% greater pregnancy rates to SexedULTRA 4 M semen compared with XY technology semen (Vishwanath 2015).

The objectives were to 1. determine the effect of the timing of insemination with SS (y‐sorted) at 12, 20, and 28 h after the onset of estrus on P/AI, sex ratio for AI, pregnancy/breeding season (P/BS), sex ratio for the breeding season, and incidence of stillbirth in beef heifers; 2. determine the effect of AI sires on P/AI, sex ratio for AI, and incidence of stillbirth in beef heifers following insemination with SS (y‐sorted); 3. elucidate the effect of differences in AI sires percentage sperm DNA fragmentation (%SDF) and timing of AI on P/AI following insemination with SS (y‐sorted).

Delaying insemination after the first onset of estrus facilitates coinciding with ovulation time, which may eliminate the AI sire differences in P/AI.

## 2 Materials and Methods

This protocol was approved by the Institutional Animal Care and Use Committee of the Washington State University (ASAF # 6987).

### 2.1 Heifers

Angus beef heifers [mean age (month ± SEM), 15.9 ± 1.1], from two locations in northwestern US, in moderate to good (5 to 7) body condition (BCS 1 to 9); 1‐emaciated; 9‐obese, (Richards et al. 1986) and with the reproductive tract score of 4 and 5 (RTS 1 to 5); 1‐acyclic, immature; 5‐cyclic mature (Kasimanickam et al. 2020; LeFever and Odde 1986) were included. Heifers were enrolled during the spring breeding programme and were blocked by age, BCS, and RTS. All heifers were dewormed using extended‐release eprinomectin to control internal and external parasites and vaccinated against bovine viral diarrhoea Types 1 and 2 viruses, infectious bovine rhinotracheitis virus, bovine parainfluenza 3, bovine syncytial virus, *Campylobacter fetus* , and five serovars of *Leptospira* (Bovi‐ Shield Gold FP5 VL5; Zoetis, Parsippany, NJ, USA) as a part of routine herd health management, at least 4 weeks prior to synchronisation. Heifers were fed mixed alfalfa or grass hay during winter, including calving and breeding time, and were then moved to a pasture, or grazed Orchardgrass, Ryegrass, and tall fescue which was supplemented with corn silage as well as a corn soybean‐meal concentrate mixture. Heifers were administered a slow‐release mineral bolus (Ultracruz Cattle Copper bolus for adults, Santa Cruz Animal Health, Dallas, TX, USA) before insemination and had ad libitum access to chelated minerals in the pasture during grazing throughout the year. Heifers were fed to meet National Research Council (NRC) recommendations (Nutrient requirements of beef cattle: eighth revised edition, 2016).

### 2.2 Estrus Synchronisation Treatment

**FIGURE 1:**
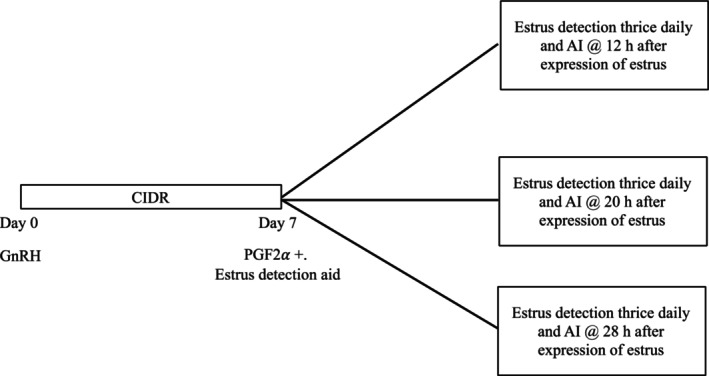
Schematic presentation of estrus synchronization treatment regimen. All heifers (*n*=913) were administered a 1.3 g progesterone intravaginal insert (CIDR, Eazi‐BreedTM CIDR Cattle Insert; Zoetis), plus 100 μg of gonadorelin diacetate tetrahydrate (GnRH, 2 mL; Factrel, Zoetis) im and 7 d later CIDR devices were removed, estrus detection aids (EstrotectTM, Western Point Inc., Apple Valley, MN, USA) were applied and 25 mg of dinoprost (PGF2 α; 2 mL; im; Lutalyse Highcon Injection; Zoetis) were administered. Heifers were observed thrice daily for standing estrus and evaluations of an estrus detector aid status were made until 96 h after PGF2 α administration. Heifers (*n* = 718) that expressed estrus were randomly assigned to receive insemination with y‐sorted sexed semen (SexedULTRA 4 M) at 12 h (*n* = 238), 20 h (*n* = 241) or 28 h (*n* = 239) after first estrus expression. Heifers (*n* = 195) that did not express estrus until 96 h were given 100 μg of GnRH im and inseminated with conventional semen concomitantly.

As per routine farm breeding management strategy, heifers (*n* = 913) were synchronised for estrus using the Select‐Synch + CIDR treatment regimen (Figure 1), but 718 heifers that expressed estrus were enrolled in the study. Briefly, heifers were administered a 1.3 g progesterone intravaginal insert (CIDR, Eazi‐BreedTM CIDR Cattle Insert; Zoetis), plus 100 μg of GnRH (gonadorelin diacetate tetrahydrate, 2 mL; im, Factrel, Zoetis). 7 days later, CIDR devices were removed, estrus detection aids (Estrotect, Western Point Inc., Apple Valley, MN, USA) were applied, and 25 mg of PGF2α (dinoprost; 2 mL; im; Lutalyse Highcon Injection; Zoetis) was administered. Heifers were observed thrice daily (approximately 8 h interval) for standing estrus, and evaluations of an estrus detector aid status were performed until 96 h after the PGF2α administration. Heifers were considered to be expressing estrus (0 h) if a cow was visually observed to stand for mounting by herd‐mates or if a heifer had an activated, lost (with mount marks) or partially activated (> 50%) estrus detection aid. Heifers that expressed estrus (*n* = 718) were randomly assigned to receive insemination at 12 (*n* = 238), 20 (*n* = 241) or 28 h (*n* = 239) after estrus was first detected. Commercial y‐sorted SS (SexedULTRA 4 M) from 3 bulls were utilised for insemination. Care was taken in assigning heifers to the respective treatment groups after detection of estrus. AI personnel (*n* = 3) and animal handlers (*n* = 11) were generally unique to each location.

In line with the farm breeding strategy, heifers that did not express estrus within 96 h (*n* = 195) were inseminated using conventional semen (CS; *n* = 6; AI sires different from those used for SS) and received 100 μg of GnRH (2 mL; im; Factrel, Zoetis). Natural service sires were introduced 2 weeks after AI.

### 2.3 Pregnancy and Calving

Transrectal ultrasonography (Sonoscape S8, Universal Imaging, Bedford Hills, New York, USA) was performed approximately 60 days after AI to determine heifers' pregnancy status. Pregnancy was confirmed by visualisation of the viable fetus. Gestational age was estimated based on sizes of embryo, amniotic vesicle, and placentomes. Sex of the calves was determined at birth. Incidences of stillbirth were recorded. Stillbirth was defined as death of calf just prior to, during, or within 48 h of parturition.

### 2.4 Sperm DNA Fragmentation by Acridine Orange Test

The microscopic acridine orange (AO) test to determine %SDF was performed as described (Apedaile et al. 2004; Tejada et al. 1984). Smears of SS samples from 3 bulls immediately (0 h) and 12 and 24 h after thawing (thawed semen maintained in 36°C) were fixed for a minimum of 3 h, washed, and air‐dried. The slides were then immersed in AO working solution [2.5 mL of 1% AO stock solution, 10 mL of 0.1 mol/L citric acid and 400 μL of 0.3 mol/L Na_2_HPO_4_.H2O (sodium hydrogen phosphate), prepared on the day of evaluation] for 5 min, rinsed with water, and wet‐mounted immediately for reading in a darkened environment. The percentage of sperm with normal (green fluorescence) and fragmented DNA (red fluorescence) was determined by rapid counting of 400 sperm under a fluorescence microscope (Leitz Wetzlar D 20) with × 1000 magnification (oil immersion lens) and excitation wavelength of 450–490 nm. For each bull, 4 replicates (200 sperm/replicate) were evaluated and averaged to determine mean %SDF.

### 2.5 Statistical Analyses

Data were analysed using a statistical software program (SAS 9.4 version, SAS Institute Inc., Cary, NC, USA). For all analyses, the differences were considered significant when *p* ≤ 0.05. As the study focused on the use of SS, and the AI sires used for CS were different from those used for SS, data from heifers (*n* = 195) inseminated with CS were excluded from the P/AI and gender ratio analysis.

Differences in mean age of heifers and BCS and time interval from estrus onset to insemination were analysed using ANOVA (PROC GLM) and with a Bartlett test used to assess homogeneity of variance. Because variances for means were heterogeneous, log10‐ or arcsine transformed data were analysed, with non‐transformed values reported. Normality was tested by PROC UNIVARIATE (Shapiro–Wilk test, *p* > 0.05). PROC GLM was used to determine the differences in percentages of heifers that expressed estrus from 12 to 24, > 24 to 48, > 48 to 72, and > 72 to 96 h.

PROC GLIMMIX, logistic regression by applying procedure GLIMMIX (METHOD = LAPLACE; ILINK = LOGIT; DIST = BINOMIAL SOLUTION ODDSRATIO) was used to determine differences in P/AI and P/BS among treatments. Fixed variables included in the analysis to determine differences in P/AI between treatments were timing of AI (12, 20 and 28 h) and bulls (*n* = 3) and treatment by bull interaction. Further, location (*n* = 2), AI sire (*n* = 3) nested in location (*n* = 2), AI personnel(*n* = 3) nested in location (*n* = 2) and animal handler (*n* = 11) nested in location (*n* = 2) were included as random variables. Fixed variable included in the analysis to determine differences in P/BS for treatment groups was timing of AI (12, 20 and 28 h). Further, location (*n* = 2), natural service bulls (*n* = 16) nested in location (*n* = 2), and animal handler (*n* = 11) nested in location (*n* = 2) were included as random variables. PROC GLM was used to determine differences in %SDF among treatment groups and bulls. PROC CORR was used to determine the correlation between percentages P/AI and sperm DNA fragmentation. In addition, PROC REG was used to determine the association between percentages P/AI and sperm DNA fragmentation.

### 2.6 Economic Analysis

Economic impact of Y‐sorted semen use in beef operation (Crude Economic Analysis) was calculated with the following assumptions.

**Table:** 

Category	Conventional semen	Sexed semen	Notes
Assumptions			
Calf mortality & weaning	Same	Same	
Cost of raising calves	Same for steer and heifer	Same	
Additional semen cost	Base: $ 20	$ 35 ($ 15 more)	
Sex ratio (male:female)	50: 50	76: 24	Based on current study
Weaning weight (lbs.)	Steer: 580 Heifer: 520	Steer: 580 Heifer: 520	
Price ($/cwt)	Steer: $ 160 Heifer: $ 150	Steer: $ 163 (premium) Heifer: $ 150	
Semen cost	$ 2000	$ 3500	Based on 100 services
Difference in semen cost		$ 1500	Sexed semen is more expensive

## 3 Results

**FIGURE 2:**
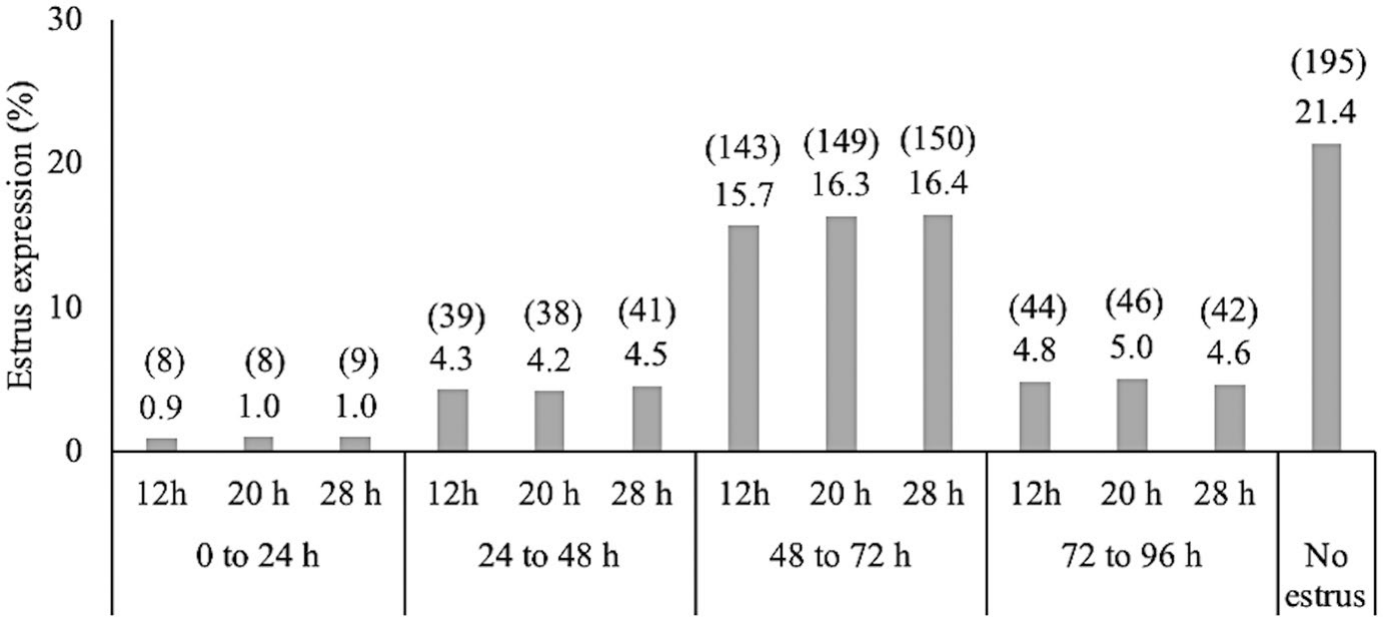
Frequency of percentages (number) of heifers that expressed estrus in each treatment group until 96 h and heifers that did not express estrus. *p* > 0.1. Refer to Figure 1 for insemination time treatment groups.

Within location, the mean age (month ± SEM) was 15.6 ± 1.1, 15.7 ± 1.2, and 15.7 ± 1.2, and BCS was 6.07 ± 0.13, 6.07 ± 0.11, and 6.10 ± 1.0 for 12, 20, and 28 h insemination treatment groups, respectively. Frequency of percentages of heifers that expressed estrus from 12 to 24, > 24 to 48, > 48 to 72, and > 72 to 96 h for each treatment group, and the percentage of heifers that did not express estrus are given in Figure 2. Mean (± SEM) interval difference between estrus onset and insemination time for treatment groups and bulls is given in Table 1. The total P/AI for heifers that expressed estrus was 50.0% (359/718). A total of 321 bulls and 38 heifer calves were born from AI with a bull: heifer sex ratio of 89%:11%. Incidence of stillbirth was 8.1% (29/359). Incidences of still birth were similar for bull and heifer calves, 7.5% (24/321) and 10.5% (4/38), respectively (*p* = 0.91). A total of 517 bulls and 160 heifer calves were born for the breeding season with a bull: heifer sex ratio of 76%:24%.

**TABLE 1:** Mean ± SEM time of insemination relative to onset of estrus for different time of AI treatment groups and bulls.

Bull	1			2			3		
Treatment§ (inseminatio*n* time from onset of estrus)	12 h	20 h	28 h	12 h	20 h	28 h	12 h	20 h	28 h
Time of insemination relative to estrus onset, h	12.6 ± 0.9	19.9 ± 0.9	28.3 ± 1.1	12.4 ± 0.8	20.2 ± 1.1	28.1 ± 1.3	12.3 ± 0.8	19.7 ± 0.9	27.8 ± 1.2

Factors influencing P/AI are given in Table 2. The P/AI were different among insemination times, 12 h—45.4% (108/238), 20 h—48.1% (116/241), and 28 h—56.5% (135/239), respectively (*p* = 0.041). The P/AI between 12 and 28 h (*p* = 0.015) differed but did not differ between 20 and 28 h (*p* = 0.067) and between 12 and 20 h (*p* = 0.546). The sex ratio (bull: heifer) following insemination at 12, 20, and 28 h was 88%:12% (95:13), 89%:11% (103:13) and 92%:8% (124:11) respectively. Incidence of stillbirth for insemination times at 12, 20, and 28 h groups was 8.3% (9/108), 8.6% (10/116), and 7.4% (10/135) respectively (*p* = 0.822).

**TABLE 2:** Factors influencing pregnancy/AI and pregnancy/breeding season following insemination with sexed semen in beef heifers.

Parameter	df^b^	*F*	*p*
Pregnancy/AI			
Insemination time treatment^a^	2	8.41	0.04
AI sire	2	3.79	0.64
Insemination time treatment by AI sire	5	7.03	0.05
Pregnancy/breeding season			
Insemination time treatment^a^	2	1.72	0.33

*Note:* Pregnancy/AI: Covariance parameter estimates: Location, 0.001459 ± 0.000964; AI sire (Location), 0.001814 ± 0.000832; AI technician (Location), 0.001341 ± 000919; Animal handler (Location), 0.002091 ± 0.000808; Residual 0.21463 ± 0.02242; Fit statistics: BIC = 717.44; −2 Res log likelihood = 714.64. Pregnancy/BS: Covariance parameter estimates: Location, 0.001982 ± 0.001081; Natural service sires (Location), 0.002127 ± 0.000991; Animal handler (Location), 0.002578 ± 0.001033; Residual 0.17742 ± 0.028442; Fit statistics: BIC = 943.31; −2 Res log likelihood = 940.19.

^a^ Refer Figure 1 for synchronisation protocol.

^b^ Degrees of freedom.

**FIGURE 3:**
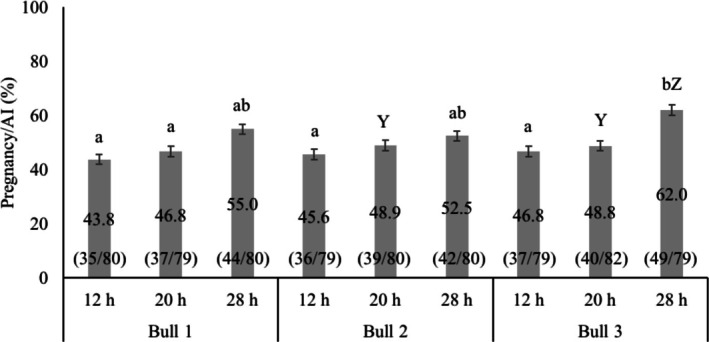
Mean ± SEM percentage pregnancy per AI for different time of AI treatment groups and bulls. §Refer to Figure 1 for insemination time treatment groups; ab, within a bull, means without a common superscript differed (*p* ≤ 0.05); YZ, means with different superscripts showed trend towards significance for P/AI (*p* < 0.1).

The P/AI among bulls was not different [bull 1, 48.5 (116/239); bull 2, 49.0% (117/239); bull 3, 52.5 (126/240); *p* = 0.635]. There was a time of insemination by bull interaction detected for P/AI (*p* = 0.05; Figure 3). The sex ratio (bull: heifer) for bulls 1, 2, and 3 was 90:10 (104:12), 88:12 (103:14) and 91:9 (115:11), respectively. Incidences of stillbirth for bulls 1, 2, and 3 were 7.8% (9/116), 8.5% (10/117), and 7.9% (10/125), respectively (*p* = 0.973).

The P/AI between locations 1 and 2 was not different, 48.9% (208/425) and 51.5% (151/293), respectively (*p* = 0.494). Bull: heifer sex ratio for locations 1 and 2 was 89:11 (185:23) and 90:10 (136:15), respectively. Incidence of stillbirth between locations 1 and 2 did not differ, 8.2% (17/208) and 7.9% (12/151), respectively (*p* = 0.938).

**FIGURE 4:**
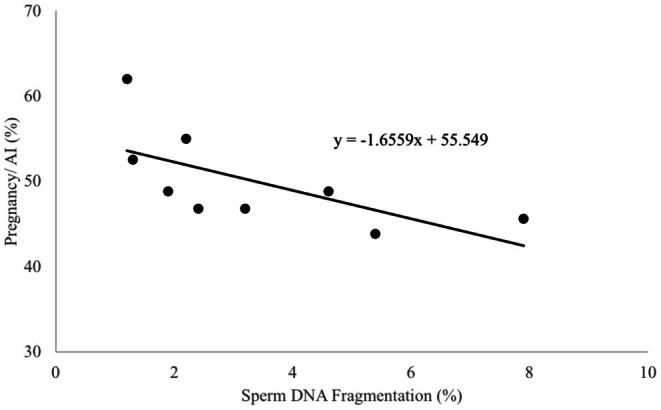
Association between percentages of pregnancy/AI and sperm DNA fragmentation. Correlation determination *r* ^2^ = 0.42; *p* = 0.05.

Sperm with normal and fragmented DNA were identified, counted, and %SDF were calculated. For bull 1, %SDF at 0 and 24 h post‐thaw did differ, 2.2% and 6.4%, respectively (*p* < 0.05; Table 3), but neither differed from 12 h, 3.2%. For bull 2, %SDF at 12 and 24 h post‐thaw were higher compared with 0 h post‐thaw, 4.6 and 7.9 vs. 1.3, respectively (*p* < 0.05). For bull 3, %SDF at 0, 12, and 24 h post‐thaw did not differ, 1.2, 1.9, and 2.4, respectively. Fold difference in %SDF among 0, 12, and 24 h post‐thaw did not differ for bull 1 and 3 but differed for bull 2 (*p* < 0.05; Table 3). There was a negative correlation between percentages of %P/AI and %SDF (r = −0.65; *p* = 0.05). The estimation of relationships between percentages of %P/AI and %SDF is given in Figure 4 (r^2^ = 0.42; *p* = 0.05).

**TABLE 3:** Mean ± SEM and fold differences in sperm DNA fragmentation index for different times of AI treatment groups and bulls.

Bull	1			2			3		
Time of sperm DNA fragmentation index evaluation post‐thaw (h)	0 h	12 h	24 h	0 h	12 h	24 h	0 h	12 h	24 h
Sperm DNA fragmentation index (%)	2.2 ± 0.9^1^	3.2 ± 0.7^12^	5.4 ± 1.9^2^	1.3 ± 0.8^1^	4.6 ± 1.2^2^	7.9 ± 1.3^3^	1.2 ± 0.4^1^	1.9 ± 0.5^1^	2.4 ± 0.8^1^
Fold change (relative to 0 h)	1.00^x^	1.45^x^	2.45^x^	1.00^x^	3.54^y^	6.08^z^	1.00^x^	1.58^x^	2.00^x^

*Note:* 123, within bull and between time of evaluations, means without a common superscript differed (*p* < 0.05). xyz, within time of evaluations and between bulls, means without a common superscript differed (*p* < 0.05).

^§^ Refer to Figure 1 for insemination time treatment groups.

Factors influencing pregnancy/breeding season are given in Table 2. The P/BS did not differ among insemination times, 12 h—91.6% (218/238), 20 h—92.5% (223/241), and 28 h—95.0% (227/239), respectively (*p* = 0.325). The P/BS among natural service bulls and locations did not differ (*p* > 0.1). The sex ratio (bull: heifer) for the breeding season for 12, 20, and 28 h treatment groups was 74:26 (161:57), 75:25 (167:56) and 79:21 (180:47), respectively.

The economic impact of Y‐sorted semen use in a beef operation is presented in Appendix 1. Based on a crude economic analysis, the use of Y‐sorted semen resulted in a profit of $ 2,670, equating to $ 26.70 per calf compared to conventional semen use.

## 4 Discussion

Utilisation of SS in the beef industry has been steadily increasing, especially in seedstock operations to produce calves of the desired sex as well as the genetics for certain traits. In the current study, delaying SS insemination to 28 h after first sign of estrus improved P/AI. The P/AI between 12 and 28 h differed (*p* < 0.05). Though the P/AI between 20 and 28 h did not differ, there was an 8.4 percentage points increase in P/AI when insemination was delayed to 28 h (*p* < 0.1).

In the current study, the gender ratio for the breeding season for 12, 20, and 28 h groups was 74:26 (161:57), 75:25 (167:56) and 79:21 (180:47), respectively. In our previous study (Kasimanickam et al. 2021), we reported that the bull: heifer gender ratio following AI with y‐sorted semen was 88:12 and 52:48 for SS and CS AI groups, respectively, with an overall bull: heifer ratio of 66:34. A bull: heifer gender ratio of 79:21 was realised after the two breeding seasons where cows were bred once by fixed time AI to SS, followed by natural mating. Another study reported 62% to 78% female calves from 3 breeding seasons following insemination once with SS (x‐sorted) (Hall et al. 2010).

A field trial assessed the use of SS (x‐sorted) in beef heifers following synchronisation with the 14‐d MGA‐PGF2 α protocol (Aubuchon 2019). The results were 95.1% estrus response, 63.4% P/AI, 87.0% P/BS with 94.3% (gender accuracy) heifers from AI and 77.7% heifers (gender skew) for the breeding season. Based on the current study, we inferred that the gender accuracy was 90% (322 bulls and 37 heifers).

Optimal AI timing for SS relative to estrous expression is different from that for CS as SS is more aged at the time of freezing due to additional handling and time required for the sorting process compared to CS (Reese et al. 2021). (Schenk et al. 2009) reported storing semen for 1 day until it can be sexed clearly depressed pregnancy rates. The fertility of SS is 75% to 90% of CS (Maicas et al. 2019; Seidel Jr. 2014). The decrease in fertility of SS could be due to its reduced lifespan in the uterus (Maxwell et al. 2004). Considering the oocyte viability in the female genital tract which is reduced after 8 h (Brackett et al. 1980) coupled with potential decreased viability of sex‐sorted sperm in the uterine tract (Maxwell et al. 2004), it is conceivable that delayed insemination with SS improved fertilisation rates by increasing the number of viable sperm available for fertilisation (Saacke 2008). The results presented in the current study suggest that SexedULTRA4M SS can be used effectively to inseminate beef heifers by delaying the time of insemination after first observed estrus expression.

Dorsey et al. (2011) assigned beef heifers to a 4 h time block based on the time from the onset of estrus (h 0) to insemination with conventional semen: 0 to 4, 4 to 8, 8 to 12, 12 to 16, 16 to 20, 20 to 24, and > 24 h to determine the effect of the time of insemination on fertility using the HeatWatch system. Pregnancies for the 4 to 24 h group (63.7%) were greater than for the 0 to 4 h (48.1%) and > 24 h groups (55.9%). Dransfield et al. (1998) using the HeatWatch system, analysed a large number of inseminations with conventional semen in dairy cattle to determine the effect of the time of insemination on fertility and found the highest fertility after AI within 4 to 12 h after the onset of standing activity (i.e., 23 to 15 h before the predicted time of ovulation). Two studies (Bombardelli et al. 2016; Stevenson et al. 1996) using activity monitors in Jersey herds found that delaying the time of insemination to 16 or more hours after reaching the standing activity threshold when using SS resulted in greater conception rates. However, Schenk et al. (2009) observed increased AI conception rates with SS when breeding was delayed 18 to 24 h after the onset of estrus in beef heifers. The greater fertility to later insemination in beef heifers [9], compared to dairy cows (Dransfield et al. 1998; Walker et al. 1996), may be related to a difference in the time of ovulation in dairy and beef cattle, 31.1 ± 0.6 h for beef heifers or cows (Dorsey et al. 2011; White et al. 2002) and 27.6 ± 5.4 h in dairy cows, respectively (DeGraaf et al. 2014). It is likely that the improved P/AI for longer intervals between the onset of estrus and the time of insemination coincides with insemination closer to ovulation. Another study that used automated activity monitors (AAM) on dairy heifers reported greater pregnancies for the 13 h time interval between SS insemination and ovulation (Macmillan et al. 2020; Walker et al. 1996). Further, P/AI in beef heifers receiving SS or CS inseminations following the 14‐d CIDR‐PG protocol did not differ when split time AI (66/90 vs. 72/96 h) was delayed 6 h (Thomas et al. 2017). In the present study, insemination using SS at 28 h in relation to the onset of estrus resulted in a greater pregnancy rate, 9.3 and 8.1 percentage points increase, compared with insemination at 12 and 20 h after estrus onset, respectively.

Dalton et al. (2001) demonstrated that the median number of accessory sperm per oocyte/ovum increased from one accessory sperm in cows that were bred 0 h after the onset of estrus to four accessory sperm when AI was performed 24 h after estrus, and this increase was associated with greater fertilisation rates (AI at 0 h = 66% vs. AI at 24 h = 82%). Saacke et al. (2000) and Saacke (2008) argued that lower quality embryos were produced when AI with non‐sorted sperm was performed closer to ovulation. Interestingly, the results presented in the current study suggest that this phenomenon may not be accurate for AI with SS. Thus, the production of poor quality embryos may be less of a problem than failures of sex‐sorted sperm to successfully fertilise an oocyte after AI, and the possibility of greater fertilisation rates with later AI intervals may occur without an increase in low‐quality embryos (Sales et al. 2011).

The interval from onset of estrus to ovulation in heifers has ranged from 25 to 32 h (Bernard et al. 1983; Larsson 1987). Macmillan (2020) study and López‐Gatius (2022) review reported that the interval from estrus onset to ovulation was 28.3 h, with a range between 16 and 46 h. When using CS, insemination within 36 h of ovulation resulted in acceptable P/AI (Colazo and Mapletoft 2017). Conversely, SS does not maintain fertility for an extended period of time in the female reproductive tract; therefore, delaying insemination closer to ovulation is recommended (Macmillan et al. 2020). In the current study, it is conceivable that the interval from AI to ovulation for 12, 20, and 28 h groups could be 18, 10, and 2 h, considering the estrus to ovulation interval is 30 h (Bernard et al. 1983; Larsson 1987). Delaying insemination with SS to within 12 h (Colazo and Mapletoft 2017) and 13 h (Macmillan et al. 2020) before ovulation was beneficial in Holstein heifers.

Variation in the fertility of individual bulls is another important factor to consider in the timing of insemination with SS. Sperm from different bulls may be differently affected by the sex‐sorting process, resulting in varying levels of sperm damage and consequently resulting in varying levels of fertility (Schenk et al. 2009). Variation in fertility in dairy bulls was greater for SS compared to CS (DeJarnette et al. 2009). Hall et al. (2017) reported pregnancy rates of 50% or greater for six sires, whereas pregnancy rates below 30% were observed for another four sires out of 19 sires used. The range of P/AI was from 19.3% to 55.6% (Hall et al. 2017). As mentioned earlier, the sex‐sorting process inflicts a variety of damage to sperm structural and functional parameters (Schenk et al. 2009). In the current study, evaluation of SDF at 0, 12, and 24 h post‐thaw did not differ for bull 1 and 3; whereas SDF differed in a time‐dependent manner among 0, 12, and 24 h post‐thaw for bull 2. Although the P/AI was not different among bulls, there was a significant interaction for time of insemination and bull. It should be noted that the P/AI for bull 1 was 4.3 and 4.3 percentage points; for bull 2, it was 14.8 and 15.2 percentage points, and for bull 3, it was 7.2 and 3.2 percentage points greater when insemination occurred 28 h after estrus onset compared with insemination at 12 and 20 h after estrus onset, respectively. The lower P/AI for heifers in the 12 and 20 h groups compared with the 28 h group was plausibly due to both an increase in the bull's post‐thaw %SDF over time and an increase in the interval from insemination to ovulation. In addition, insemination of SS close to ovulation may eliminate bull‐to‐bull variation in P/AI by reducing the difference in %SDF post‐thaw over time and sperm lifespan in the female reproductive tract.

Interestingly, similar results were reported in a field trial evaluating the effect of the timing of insemination relative to the onset of estrus on pregnancy rates using SS (x‐sorted) in beef heifers (Aubuchon et al. 2023). Heifers were subjected to a 14‐d MGA‐PGF2 α synchronisation protocol and observed every 4 h for estrus. The P/AI for heifers inseminated at 12 to 15.9 h (*n* = 57), 16.5 to 21.0 h (*n* = 33) and 21.4 to 27.5 h (*n* = 28) after the onset of estrus were 51.4%, 56.3%, and 75.9%, respectively. The P/AI for heifers inseminated between 21.4 and 27.5 h was greater (*p* > 0.05), 24.5 percentage points, compared to the heifers inseminated between 12 to 15.9 h. Though P/AI for heifers inseminated between 21.4 and 27.5 h and 16.5 to 21.0 h was not significant, there was a 19.6 percentage points increase in P/AI for the 21.4 and 27.5 h group.

In the current study, an estrus detection patch was used to determine the time of estrus onset. The adoption of an automated activity monitor (AAM; SCR eSense ear tag) provides new opportunities in commercial cattle operations for the application of AI with SS technology. Macmillan et al. (2020) evaluated the performance of the AAM to detect estrus behaviour and estrus detection patches (Estrotect) with thrice daily estrus detection. In that study, the results showed that the sensitivity (ability to correctly identify heat events, true positive estrus) was 91.0% with 8.0% false positive and 8.0% false negative rates for the AAM. The positive predictive value (true estrus events/all estrus events) of the AAM system was 83.5% of the estrus detection patch. In total, 21 heifers were bred based on estrus detection patches (false negative heats), with 19 becoming pregnant. Therefore, the adoption of estrus alert patches detects estrus equally or more than the AAM.

For wide‐spread acceptance, insemination with SS must achieve similar P/AI comparable to CS and must be appropriate for use in FTAI protocols. Recent studies are focusing on the application of SS insemination following the FTAI protocol (Crites et al. 2018; Ketchum et al. 2021; Oosthuizen et al. 2021; Schenk et al. 2009; Thomas et al. 2017). Though increased adoption of SS insemination in the beef industry is reliant on the development of FTAI protocols that do not require the labour for estrus detection, new technology such as AAM with better understanding could be utilised for efficient application.

Beef operations that achieve high P/AI, maintain effective estrus detection, and exhibit substantial gender‐based differences in calf value may benefit economically from the strategic use of sexed semen. In such systems, targeted application of Y‐sorted semen can enhance profitability by increasing the proportion of male calves, which typically exhibit superior growth performance (up to 60 lbs.) and carcass traits compared to heifers (Hall and Glaze Jr 2013; Hall and Glaze Jr 2014). For example, steer calves generally weigh approximately 22.8 kg more than heifer calves at weaning (Dinkel et al. 1992). The 10, 5‐year, and for 2024 steer‐heifer price differences for 500 to 600 lbs. weight class were calculated using data from USDA compiled by LMIC (https://lmic.info/resources Accessed July 19, 2025) as 24.14, 25.99, and $ 35.09, respectively.

However, the widespread adoption of sexed semen in beef production remains limited. Reduced conception rates compared to conventional semen and the relatively high cost of sex‐sorted products often make them less economically viable, particularly in low‐fertility or extensive production systems. Previous studies have thoroughly explored the potential applications of sexed semen in both the beef and dairy industries (Hohenboken 1999; Seidel Jr. 2003). Despite these limitations, sexed semen can be integrated effectively into strategic breeding programmes. One approach involves using sexed semen to produce heifer calves from sires selected for superior maternal traits, thereby improving the quality of replacement females. These females can then be bred using Y‐sorted semen from sires with high terminal performance, maximising the value of male offspring. This two‐tiered strategy not only enhances heterosis and breed complementarity but also aligns genetic selection with specific production goals, ultimately improving both productivity and profitability.

In the current study, a crude economic analysis of Y‐sorted semen use in a beef operation demonstrated a net profit of $ 2,670, equivalent to $ 26.70 per calf when compared to conventional semen use. These findings suggest that, under the right management conditions, Y‐sorted semen can contribute positively to economic returns in beef operations when applied with careful planning and clear production objectives.

## 5 Conclusion

Delaying of insemination with SS up to 28 h after the onset of estrus resulted in improved P/AI. Delayed SS insemination that occurred close to ovulation resulted in greater P/AI. This improved P/AI due to delayed insemination with SS was more evident for bulls undergoing increased post‐thaw sperm DNA fragmentation over time.

## Author Contributions

These authors contributed equally to this work.

## Conflicts of Interest

The authors declare no conflicts of interest.

## Data Availability

The data that support the findings of this study are available from the corresponding author upon reasonable request.

## Appendix 1

Economic Impact of Y‐Sorted Semen Use in Beef Operation (Crude Economic Analysis).

Net Return.

**Table:**

Calf Type	No. of calves	Weaning weight (lbs.)	Price ($/cwt)	Net return
Conventional semen				
Steer	50	580	$ 160	50 × 580 × (160/100) = $ 46,400
Heifer	50	520	$ 150	50 × 520 × (150/100) = $ 39,000
Total	—	—	—	$ 85,400
Sexed semen				
Steer	76	580	$ 163	76 × 580 × (163/100) = $ 71,850
Heifer	24	520	$ 150	24 × 520 × (150/100) = $ 18,720
Total	—	—	—	$ 89,570

Profit

**Table:**

Item	Amount ($)
Net return (sexed)	$ 89,570
Net return (conventional)	$ 85,400
Difference	$ 4170
Extra semen cost	$ 1500
Profit	$ 2670
Profit per calf	$ 26.70
